# In Reply to Gürdoğan and Altay

**DOI:** 10.4274/balkanmedj.galenos.2019.2019.5.140-reply

**Published:** 2019-08-22

**Authors:** Marina Ruxandra Otelea, Adrian Streinu-Cercel, Cristian Băicus, Maria Nitescu

**Affiliations:** 1 Carol Davila University of Medicine and Pharmacy, Bucharest, Romania; 2 Institute for Infectious Diseases ‘Matei Bals’, Bucharest, Romania; 3 Clinical Hospital Colentina, Bucharest, Romania

To the Editor,

We would like to thank you for this letter that gives us an opportunity to further comment on the results of our study (1).

In the article, we emphasized the importance of the adipokine profile, as a whole and not a single biomarker, as the main conclusion of our research. In view of the present data, the leptin to adiponectin ratio is a strong and affordable candidate, but we cannot disregard the potential of the large number of adipokines secreted by unhealthy adipocytes to be better markers. Moreover, we are aware that gender differences have been reported with respect to adipokine secretion; therefore, the correlations that we reported were checked for gender influence. The correlation was maintained, regardless of gender.

However, this does not mean that the individual components, i.e., the leptin and adiponectin plasma values, did not differ between men and women in our study. In accordance to the article cited by Dr. Gürdogan and Dr. Altay, we found both leptin and adiponectin to be significantly higher in women than that in men (2). However, contrary to the previously mentioned study (1), the leptin to adiponectin ratio was similar in both the sexes (Kruskal–Wallis, ANOVA, p=0.845, H=0.038). Similarly, no significant gender differences were reported by other authors with respect to the leptin to adiponectin ratio and the incidence of the metabolic syndrome (3) in cross sectional studies.

The different reference values in women and men are probably justified by the hormonal secretions and distribution of visceral versus subcutaneous fat (4,5). In prospective studies, this difference leads to gender-specific cut-offs of the leptin to adiponectin ratio for the prediction of risk (6) as well as prediction of the regression of the metabolic syndrome in high-risk individuals (7). Our study was not a longitudinal one and did not analyze such differences; however, if it would have been, the sex difference in the adipokine profile would have been a part of the analysis.

There are definitely more questions to be answered about the best biomarker profile of the unhealthy adipose tissue (8), and we agree that distinguishing the gender and age categories will better stratify the risk.
